# VK-2019 enhances gemcitabine’s antitumor activity in EBV-positive nasopharyngeal carcinoma by upregulating PRODH expression

**DOI:** 10.1038/s41420-026-03247-z

**Published:** 2026-07-08

**Authors:** Chengxun Li, Dantong Gu, Yingzhu Li, Li Wang, Hongmeng Yu

**Affiliations:** 1 Department of Otolaryngology, Eye & ENT Hospital, Fudan University, Shanghai, 200030 China; 2 Department of Clinical Laboratory, Eye & ENT Hospital, Shanghai Medical College, Fudan University, Shanghai, China

**Keywords:** Drug discovery, Head and neck cancer

## Abstract

Nasopharyngeal carcinoma (NPC) is an Epstein-Barr virus (EBV)-driven malignancy, making the viral protein EBNA1 a pivotal therapeutic target. This study evaluates VK-2019, a clinical-stage EBNA1 inhibitor, for its potential to sensitize NPC cells to chemotherapy. We report that VK-2019 synergistically enhances gemcitabine efficacy, reducing the IC50 by nearly 30-fold in EBV-positive C666-1 cells. Transcriptomic profiling combined with in vitro and in vivo models reveals a distinct mechanism: VK-2019 inhibition of EBNA1 downregulates c-MYC, thereby derepressing its downstream target, Proline Dehydrogenase (PRODH). Furthermore, we demonstrate that gemcitabine independently upregulates PRODH via p53 pathway activation. This dual induction of PRODH drives enhanced anti-tumor cytotoxicity. These results provide a prominent preclinical rationale for evaluating VK-2019 in combination regimens and offer a potential therapeutic strategy for patients with EBV-associated nasopharyngeal carcinoma.

## Introduction

Nasopharyngeal carcinoma (NPC) is a malignant neoplasm strongly associated with Epstein-Barr virus (EBV) and exhibits a high incidence in southern China, Southeast Asia, and North Africa [1]. EBV mediates extensive epigenetic reprogramming in host cells through the expression of multiple viral oncogenes—such as EBNA1, LMP-1, and LMP-2A—and non-coding RNAs, including EBERs and BART microRNAs [2]. These viral factors modulate key cancer-related signaling pathways, such as those governing sustained proliferative capacity, resistance to cell death, immune evasion, and genomic instability [3]. Consequently, the development of EBV-specific targeted therapeutic strategies holds significant scientific value and promising clinical potential.

Currently, the management of nasopharyngeal carcinoma primarily depends on radiotherapy, chemotherapy, and surgical intervention, with a notable absence of effective molecularly targeted therapies [4]. For patients with advanced disease and distant metastasis, even when treated with concurrent chemoradiotherapy or combination regimens, the 5-year survival rate remains approximately 50%, highlighting the urgent need for more effective and safer therapeutic options [5]. However, progress in the development of Epstein-Barr virus (EBV)-targeted therapeutics has been limited. This is partly due to the fact that most EBV infections are self-limiting, with many individuals remaining asymptomatic or recovering spontaneously [6]. Moreover, EBV exists in two distinct infection phases: lytic and latent. During latent infection, the virus produces minimal viral particles and instead relies on a small set of essential proteins to maintain genomic persistence and drive oncogenesis, rendering conventional antiviral strategies ineffective [7, 8].

In recent years, advances in biotechnology and immunotherapy have facilitated the transition of EBV-targeted therapies into clinical investigation. Tabelecleucel, an allogeneic Epstein-Barr virus (EBV)-specific T-cell immunotherapy, can recognize and eliminate EBV-positive cells in a human leukocyte antigen (HLA)-restricted manner [9]. Furthermore, chimeric antigen receptor T-cell (CAR-T) therapy targeting EBV antigens has received FDA approval for phase II clinical trials in patients with recurrent or metastatic EBV-positive nasopharyngeal carcinoma [10]. Additionally, studies have demonstrated that the combination of chemotherapy and interferon-beta significantly enhances therapeutic efficacy in nasopharyngeal carcinoma. These developments underscore the clinical significance of targeting EBV in the treatment of EBV-associated malignancies [11]. Among viral proteins, EBNA1—a central protein in the EBV life cycle—plays a pivotal role in viral genome replication and long-term episomal maintenance, while also contributing to the modulation of host cell oncogenesis, thereby representing a promising therapeutic target [12]. In 2019, Messick et al. identified a small-molecule inhibitor, VK-2019, through high-throughput screening and structural optimization, which specifically disrupts EBNA1 function [13]. Clinical evaluations have shown that VK-2019 exhibits favorable safety profiles in patients with advanced EBV-positive nasopharyngeal carcinoma, achieving micromolar plasma concentrations and demonstrating measurable target-specific biological activity in tumor tissues [14]. However, while the clinical-stage inhibitor VK-2019 has demonstrated safety and target engagement, its efficacy as a monotherapy remains limited in patients with advanced NPC [14–16]. Our study addresses this critical gap by providing a translational rationale for combination therapy.

In this study, considering that cisplatin, docetaxel, and gemcitabine represent the current standard-of-care for nasopharyngeal carcinoma, we first performed a comparative evaluation to identify which agent most effectively synergizes with VK-2019. Based on the robust chemosensitization observed specifically with gemcitabine, we subsequently investigated the underlying molecular mechanisms driven by the EBNA1-c-MYC-PRODH axis. The results demonstrated that VK-2019 significantly enhanced the antitumor efficacy of gemcitabine against EBV-positive nasopharyngeal carcinoma cells. Through transcriptome sequencing analysis, complemented by in vitro and in vivo model validation, we further revealed that VK-2019 acts synergistically with gemcitabine to activate the p53 signaling pathway, upregulate PRODH expression, promote tumor cell apoptosis, and improve therapeutic outcomes. This study not only clarifies the molecular mechanisms underlying the interaction between VK - 2019 and chemotherapy drugs but also offers a theoretical and translational basis for the clinical application of targeted therapies for EBV - positive tumors.

## Results

### VK-2019 enhances the inhibitory activity of gemcitabine on EBV positive nasopharyngeal carcinoma cells

**Fig. 1:**
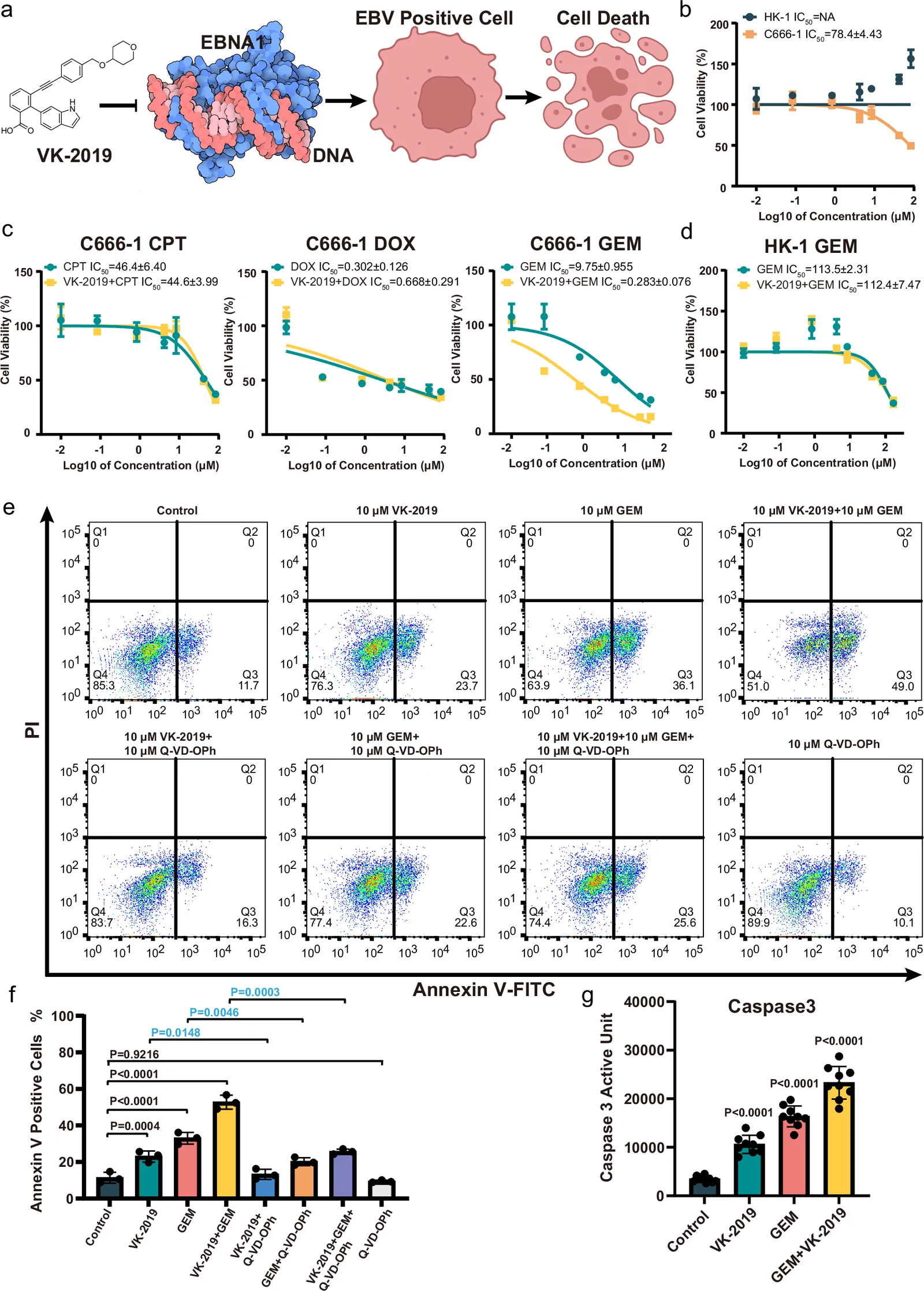
VK-2019 enhances the inhibitory effect of gemcitabine on Epstein-Barr virus-positive tumor cells. **a** The pharmacological mechanism of VK-2019. **b** Inhibition curves of VK-2019 on C666-1 (Epstein-Barr virus-positive tumor cells) and HK-1 (Epstein-Barr virus-negative tumor cells) cells. **c** Inhibition curve of VK-2019 combined with other chemotherapy drugs on C666-1 cells. The values shown in the legend are IC_50_ values (mean ± SD, *n* = 3 biological replicates). IC_50_, the half maximal inhibitory concentration(μmol/L), was obtained from GraphPad Prism 10.4. The working concentration of VK-2019 is 10 μM. CPT, Cisplatin; DOX, Docetaxel; GEM, Gemcitabine. **d** Inhibition curve of VK-2019 combined with Gemcitabine on HK-1 cells. **e** The inhibitory effect of VK-2019 and gemcitabine on C666-1 cells analyzed by flow cytometry. **f** Statistical data of cell death from **e (***n* = 3 biological replicates**)**. **g** Enzyme activity measurement of caspase 3 in C666-1 cells (*n* = 9 biological replicates). The data in panels **f** and **g** are presented as mean ± SD. Comparisons with the control group were performed using Dunnett’s multiple comparisons test (black *P*-values), and pairwise comparisons between groups were conducted using an unpaired t-test (blue *P*-values).

VK-2019, a small molecule inhibitor, suppresses the proliferation of EBV-positive tumor cells by inhibiting EBNA1 [14], thereby inducing cellular apoptosis (Fig. 1a). The results of this study demonstrate that VK-2019 significantly inhibits EBV-positive nasopharyngeal carcinoma cells (C666-1) [17], with minimal inhibitory effect observed in EBV-negative nasopharyngeal carcinoma cells (HK-1) (Fig. 1b) [18]. Cisplatin (CPT), docetaxel (DOX), and gemcitabine (GEM) are commonly used chemotherapeutic agents in the treatment of nasopharyngeal carcinoma [3]. The combination with VK-2019 resulted in a greater than 30-fold reduction in the IC_50_ value of GEM in C666-1 cells (9.75 vs. 0.283) (Fig. 1c), whereas no significant change in IC_50_ was observed in HK-1 cells (113.5 vs. 112.4) (Figs. 1d, S2). To identify an optimal combination regimen, we evaluated VK-2019 alongside three standard chemotherapeutic agents (CPT, DOX, and GEM) (Fig. 1c). Among these, only the combination with gemcitabine resulted in a substantial ~30-fold decrease in IC_50_ in EBV-positive C666-1 cells. To further validate the findings, we evaluated the combination efficacy in SNU-719 cells, a naturally EBV-positive gastric cancer model with wild-type p53 [19]. Consistent with our observations in C666-1 cells, VK-2019 (10 μM) significantly potentiated the anti-tumor activity of gemcitabine in SNU-719 cells, leading to a marked shift in the IC_50_ (Fig. S2c). To formally establish the pharmacological interaction between VK-2019 and GEM, we performed a comprehensive dose-matrix analysis (Fig. S3). The mean synergy scores for ZIP (34.36), Bliss (34.59), Loewe (26.82), and HSA (32.77) were all significantly above 10 (*P* < 0.0001), confirming a highly potent synergistic interaction [20]. This formal modeling validates that the enhanced antitumor activity observed is a result of true pharmacological synergy rather than a simple additive effect.

Flow cytometry analysis demonstrated that both GEM and VK-2019 induce apoptosis in C666-1 cells, with their combination exhibiting a more pronounced pro-apoptotic effect (Fig. 1e, f). Furthermore, Q-VD-OPh, a broad-spectrum caspase inhibitor [21], significantly attenuated apoptosis induced by either VK-2019 or GEM (Fig. 1e, f), indicating that the apoptotic effects of these compounds are mediated through caspase-dependent pathways. The caspase-3 activity assay results further confirm that both GEM and VK-2019 are capable of activating caspase-3, with a more pronounced activation observed when the two compounds are used in combination (Fig. 1g). These findings suggest that GEM and VK-2019 not only effectively induce apoptosis in cells but also exert a stack effect when administered together.

### VK-2019 enhances GEM’s ability to resist C666-1 cells by upregulating PRODH

**Fig. 2:**
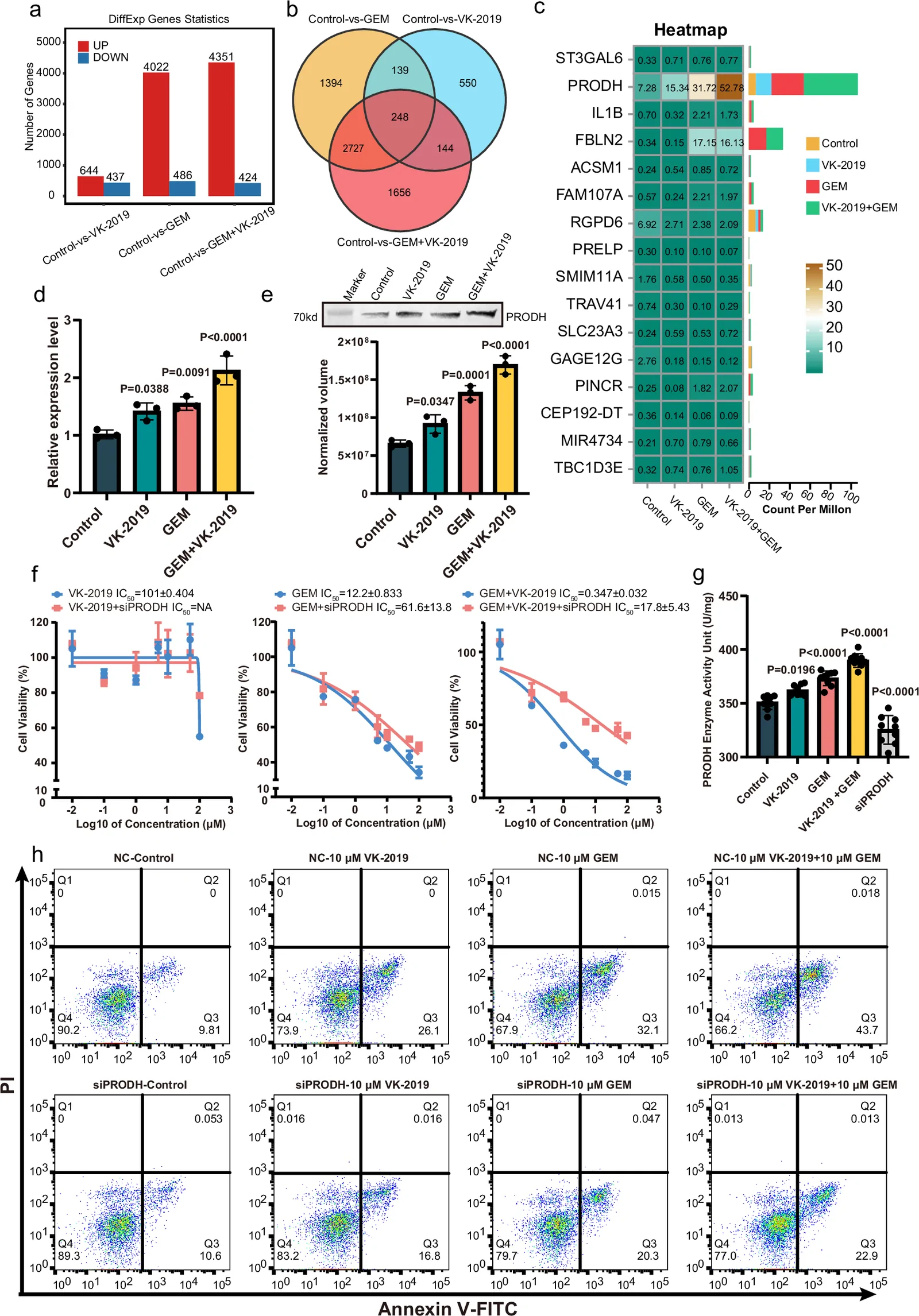
Transcriptome sequencing analysis of the mechanism underlying the enhancement of GEM’s antitumor efficacy by VK-2019 for C666-1 cells. **a** Bar chart for statistical analysis of differentially expressed genes. **b** Venn diagram for statistical analysis of differentially expressed genes. **c** Heatmap of differentially expressed genes in each group (*n* = 3 biological replicates). The data in the square represents CPM (Counts Per Million) values. **d** QPCR results of PRODH in each group (*n* = 3 biological replicates). **e** Western Blot results of PRODH in each group (*n* = 3 biological replicates). The above figure shows PRODH western blotting. Uncropped western blotting and total protein are showed in Fig. S9. The quantification of western blot (Bar chart below) was normalized with total protein by ImageLab 6.0 software, and the control sample as a reference. **f** Inhibition curve of VK-2019 combined with Gemcitabine on PRODH knockdown cells. The values shown in the legend are IC_50_ values (mean ± SD, *n* = 3 biological replicates). **g** Enzyme activity measurement of PRODH in C666-1 cells (*n* = 9 biological replicates). The data in panels **d,** **e** and **g** are presented as mean ± SD. **h** The inhibitory effect of VK-2019 and gemcitabine on PRODH knockdown cells analyzed by flow cytometry.

To investigate the mechanism by which VK-2019 enhances the anti-tumor effect of GEM, bulk RNA sequencing was performed. The transcriptome sequencing analysis reveals that the number of differentially expressed genes in the VK-2019 group is lower than that in the GEM group, with a greater proportion of upregulated genes relative to downregulated genes observed in the GEM group (Figs. 2a, S4). The intersection analysis of differentially expressed genes across groups identified a total of 248 commonly shared differentially expressed genes (Fig. 2b). Among these, some common differentially expressed genes exhibiting consistent expression changes in the VK-2019, GEM, and VK-2019/GEM groups were further analyzed. Heatmap visualization revealed that PRODH exhibited a significant and consistent upregulation across all treatment groups, with the most pronounced expression changes observed in the VK-2019/GEM group compared to the GEM and VK-2019 groups (Fig. 2c). The results of qPCR and Western blot analyses demonstrated that VK-2019 and GEM effectively upregulate PRODH expression at both the mRNA and protein levels (Fig. 2d, e). To confirm VK-2019’s effect on PRODH is on-target via EBNA1 inhibition, we knocked down EBNA1 with siRNA in C666-1 cells. As shown in Fig. S5, EBNA1 protein levels dropped significantly, and PRODH upregulation, mirroring VK-2019 treatment, was recapitulated. This provides direct genetic evidence that EBNA1 negatively regulates PRODH.

To further validate the role of PRODH in mediating anti-tumor effects, a PRODH-knockdown C666-1 cell line was established via siRNA electroporation. Following validation by quantitative PCR and Western blot analysis (Fig. S6), siPRODH3 was selected as the sequence for subsequent experiments in this study. Knockdown of PRODH in C666-1 cells significantly reduced cellular sensitivity to both VK-2019 and GEM, as evidenced by IC_50_ values increasing several- to tenfold (Fig. 2f). The vitality assay of PRODH further confirms that VK-2019 and GEM can significantly enhance PRODH enzymatic activity (Fig. 2g). The above data indicates that the expression level and activity of PRODH are crucial for the inhibitory effect of VK-2019 and GEM on C666-1 cells.

**Fig. 3:**
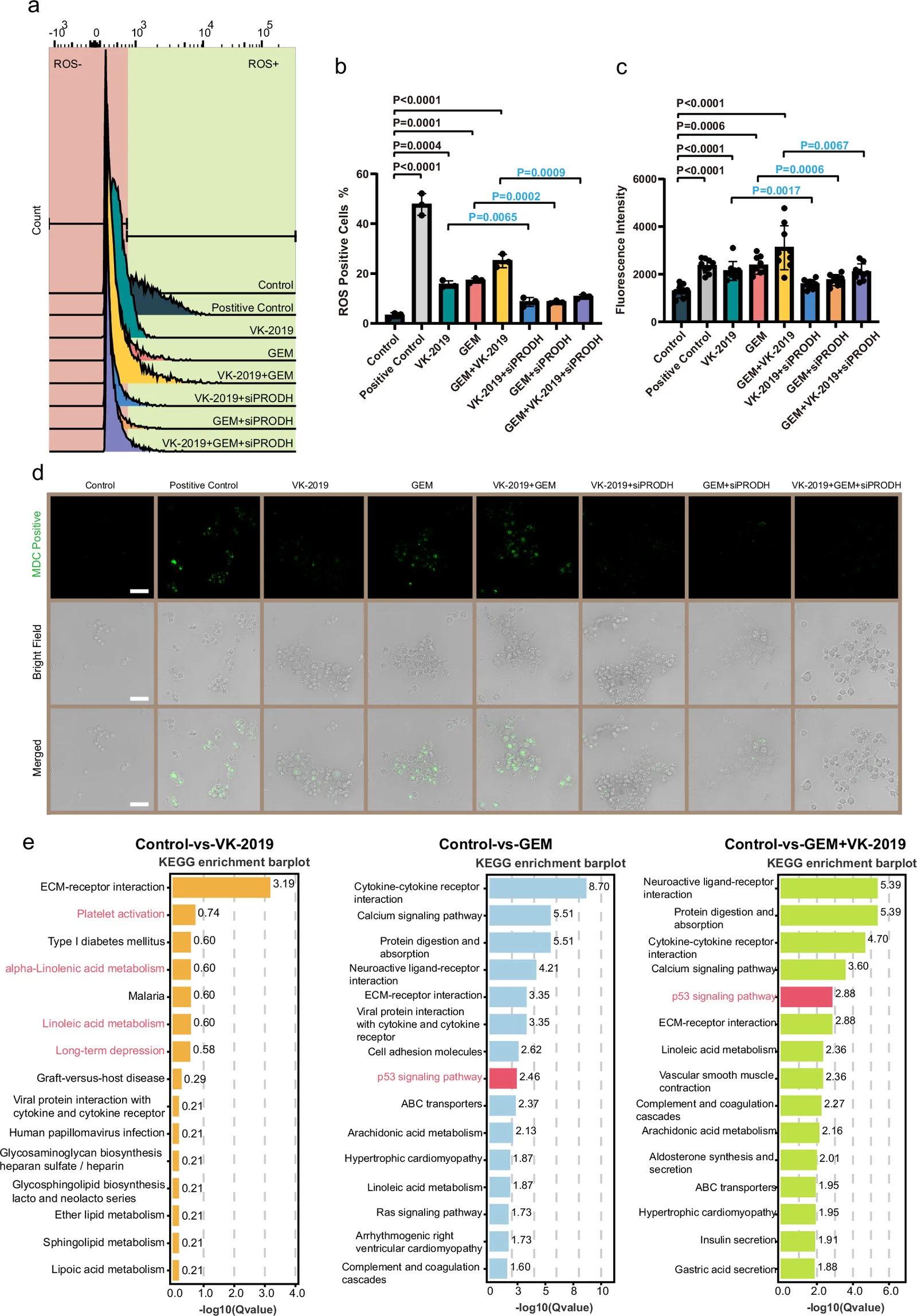
VK-2019 upregulates PRODH, increasing ROS levels and autophagy in C666-1 cells. **a** Quantitative analysis of ROS in C666-1 cells after co incubation with VK-2019 and GEM. **b** Statistical data of ROS positive cells from **a (***n* = 3 biological replicates**)**. **c** Fluorescence detection of autophagy induced by VK-2019 in C666-1 cells (*n* = 9 biological replicates). The data in panels **b** and **c** are presented as mean ± SD. Comparisons with the control group were performed using Dunnett’s multiple comparisons test (black *P*-values), and pairwise comparisons between groups were conducted using an unpaired t-test (blue *P*-values). **d** Laser confocal imaging VK-2019 induces autophagy in C666-1 cells. Scale bars, 50 µm. MDC is a fluorescent probe for detecting cellular autophagy. The positive control was cells treated with Earle’s Balanced Salt Solution (EBSS) for 1 h. **e** KEGG enrichment of differentially expressed genes in each group.

PRODH is an enzyme that catalyzes the initial step of proline degradation, a process accompanied by the generation of reactive oxygen species (ROS) and/or adenosine triphosphate (ATP) [22, 23]. Consequently, PRODH activity is closely associated with the induction of cell apoptosis and autophagy [23]. The flow cytometry analysis demonstrated that PRODH knockdown in C666-1 cells attenuated the apoptotic induction mediated by VK-2019 and GEM (Fig. 2h), indicating a critical role of PRODH in the apoptosis of C666-1 cells triggered by these compounds. Meanwhile, VK-2019 and GEM can significantly upregulate ROS in C666-1 cells, and PRODH knockdown reduced the proportion of cells exhibiting ROS upregulation (Fig. 3a, b). To further substantiate the definitive role of PRODH as the central convergent mediator in our model, we performed a functional over-expression experiment. Western blot analysis confirmed the successful ectopic over-expression of PRODH in C666-1 cells (Fig. S7a). Notably, the forced expression of PRODH was sufficient to trigger a significant increase in ROS production (Fig. S7b, c) and a concomitant elevation in the apoptotic cell population (Fig. S7d).

Qualitative and quantitative assessment of VK-2019- and GEM-induced autophagy in C666-1 cells was performed using monodansylcadaverine (MDC) staining. Confocal laser microscopy and the fluorescence microplate reader results demonstrated that both VK-2019 and GEM effectively induce autophagy, an effect that is attenuated following PRODH knockdown (Fig. 3c, d). To determine whether autophagic structures accumulated due to increased autophagosome formation or impaired lysosomal degradation, we measured autophagic flux. As shown in Fig. S8, VK-2019 and GEM each increased LC3B-II and p62 levels; adding the lysosomal inhibitor bafilomycin A1 caused significantly greater accumulation of both markers than bafilomycin A1 alone. Therefore, VK-2019 and GEM can effectively induce apoptosis and autophagy in C666-1 cells through the upregulation of PRODH.

### VK-2019 and GEM upregulate PRODH in C666-1 cells via different signaling pathways

**Fig. 4:**
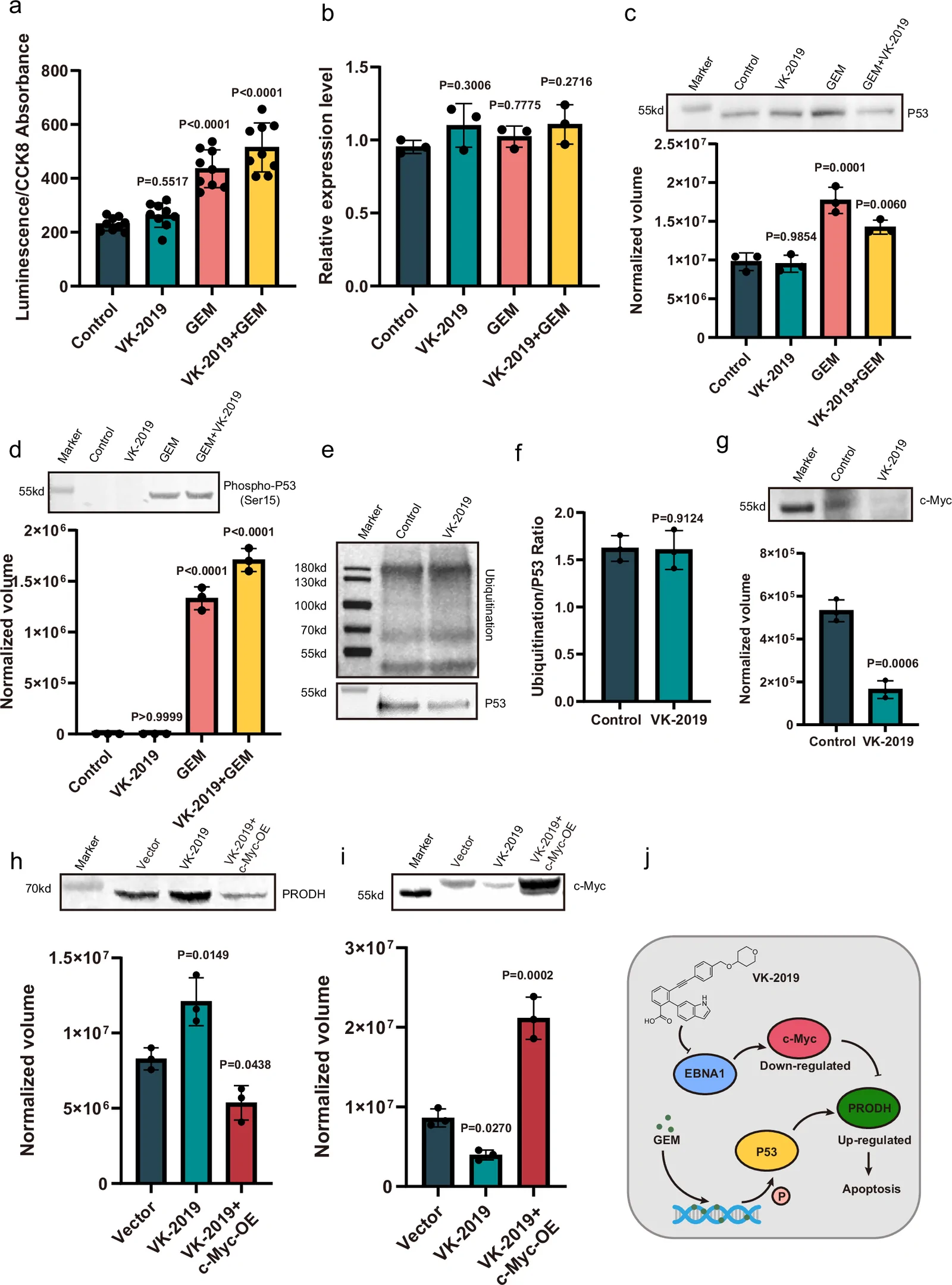
VK-2019 upregulates P53 transcriptional activity. **a** Luciferase assay used to detect VK-2019’s regulation of p53 transcriptional activity (*n* = 9 biological replicates). The data was normalized based on the cell viability values detected by CCK8, with the Control group as the reference. **b** QPCR results of P53 in each group (*n* = 3 biological replicates). **c** Western Blot results of P53 in each group (*n* = 3 biological replicates). Uncropped western blotting and total protein are showed in Fig. S11. **d** Western Blot results of Phospho-P53 (Ser15) in each group (*n* = 3 biological replicates). Uncropped western blotting and total protein are showed in Fig. S12. **e** Western Blot results of ubiquitinated P53 (*n* = 3 biological replicates). Uncropped western blotting is showed in Fig. S13. **f** The quantification of western blot from **e**. **g** Western Blot results of c-Myc in each group (*n* = 3 biological replicates). Uncropped western blotting and total protein are showed in Fig. S14. **h** Western Blot results of PRODH (*n* = 3 biological replicates). Uncropped western blotting is showed in Fig. S15. **i** Western Blot results of c-Myc (*n* = 3 biological replicates). Uncropped western blotting is showed in Fig. S15. Data are presented as mean ± SD. Statistical significance versus the control group was determined by unpaired Student’s t-tests **f, g** or Dunnett’s multiple comparisons tests **a–d, h, i**. Western blot bands were quantified using ImageLab 6.0 and normalized to total protein. **j** Schematic diagram of the mechanism by which VK-2019 enhances the anti-tumor activity of gemcitabine.

Through KEGG enrichment analysis of transcriptome data, the P53 signaling pathway was significantly enriched in the GEM group (Fig. 3e). To further investigate whether the transcriptional activity of P53 is upregulated by VK-2019 and GEM, the luciferase reporter gene was electroporated into C666-1 cells. The results showed that co incubation of C666-1 cells with GEM significantly upregulated the transcriptional activity of P53 (Fig. 4a). However, the qPCR results demonstrated that neither VK-2019 nor GEM significantly upregulated the mRNA expression level of P53, whereas western blot analysis revealed that GEM significantly increased the protein expression of P53 (Fig. 4b, c). This suggests that GEM enhance the transcriptional activity of P53 independently of P53 mRNA upregulation. Some studies have demonstrated that GEM can be integrated into DNA as an aberrant sequence, leading to enhanced phosphorylation of P53 at Ser15, thereby increasing the protein’s stability and transcriptional activity [24, 25]. The western blot results further confirmed that GEM increased the protein levels of phosphorylated P53 at Ser15 in C666-1 cells (Fig. 4d), indicating that GEM enhances P53 transcriptional activity through promotion of its phosphorylation. Certain research findings have indicated that EBNA1 is capable of upregulating the ubiquitination level of P53, consequently facilitating the proliferation of tumor cells [26, 27]. Through the immunoprecipitation of the P53 protein, the ubiquitination level of P53 was measured, and the outcomes demonstrated that VK-2019 did not lead to a significant increase in the ubiquitination level of P53 (Fig. 4e, f). To determine whether the induction of PRODH by GEM is mediated via the p53 signaling pathway, we employed the p53-specific transcriptional inhibitor, Pifithrin-α, in C666-1 cells. Western blot analysis revealed that GEM significantly upregulated PRODH protein levels (Fig. S10). Crucially, pre-treatment with Pifithrin-α significantly blunted this GEM-induced upregulation (Fig. S10), confirming that PRODH is a downstream functional target of p53 activation in this context.

The findings from transcriptome sequencing indicated a significant down - regulation of PLA2 regulated by c-Myc within the signaling pathways (highlighted in red) (Fig. 3e, Supplement Table 1). This suggests that the transcriptional function of c-Myc is influenced [28]. The outcomes of Western blot analysis indicated that VK-2019 can substantially downregulate the expression of c-Myc (Fig. 4g), a crucial transcriptional repressor of PRODH expression [29]. To confirm that c-Myc suppression causes PRODH induction, we rescued c-Myc expression in VK-2019-treated C666-1 cells (Fig. 4h**, i**). Western blotting showed that VK-2019 reduced c-Myc protein and increased PRODH; ectopic c-Myc expression reversed this PRODH upregulation. The research studies have verified that EBNA1 can upregulate the expression of c-Myc via diverse pathways, thereby facilitating the proliferation and dedifferentiation of tumor cells [30]. Therefore, GEM facilitates the phosphorylation of P53. Meanwhile, VK-2019 downregulates c-Myc and mitigates its transcriptional inhibitory effect on PRODH. Ultimately, they jointly promote the upregulation of PRODH, which leads to the apoptosis of EBNA1-positive tumor cells (Fig. 4j).

### VK-2019 potentially exacerbates the hematological toxicity associated with GEM

**Fig. 5:**
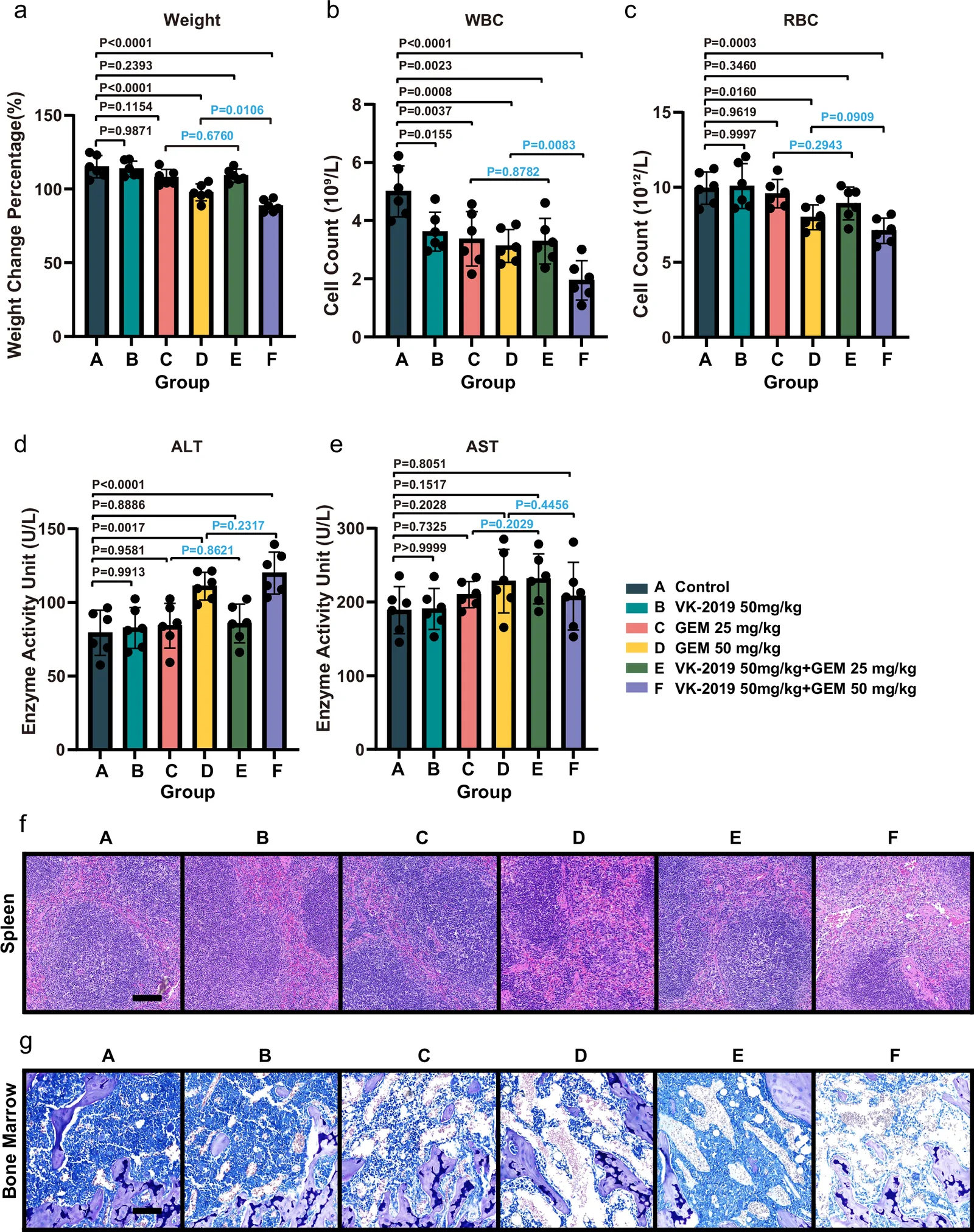
Toxicity analysis of VK-2019 combined with gemcitabine (*n* = 6 biological replicates). **a** Percentage change in body weight of mice after medication. **b** White blood cell (WBC) count statistics. **c** Red blood cell (RBC) count statistics. **d** Statistical analysis of alanine aminotransferase (ALT) detection. **e** Statistical analysis of aspartate aminotransferase (AST) detection. The data are presented as mean ± SD (*n* = 6). Comparisons with the control group were performed using Dunnett’s multiple comparisons test (black *P*-values), and pairwise comparisons between groups were conducted using an unpaired t-test (blue *P*-values). **f** H&E staining of spleen tissue. Scale bars, 100 µm. **g** Wright-Giemsa staining of bone marrow tissue. Scale bars, 100 µm.

To further investigate the in vivo toxicity of VK-2019 in combination with GEM, multiple dosing regimens were evaluated in this study. Mouse body weight monitoring revealed that high-dose GEM alone induced weight loss, and the combination of high-dose GEM with VK-2019 resulted in a significantly greater reduction in body weight (Fig. 5a). This suggests that VK-2019 may potentiate the in vivo toxicity of GEM at higher doses. However, no significant weight loss was observed when VK-2019 was combined with low-dose GEM (Fig. 5a). Clinical data shows that VK-2019 exhibits certain blood toxicity when administered at high doses [14]. In this study, the white blood cell count results demonstrated that the VK-2019 group exhibited a significantly lower level compared to the control group (Fig. 5b). While VK-2019 did not aggravate the reduction in white blood cells in the low-dose GEM group, it exerted a significant exacerbating effect in the high-dose GEM group (Fig. 5b). The platelet count results demonstrated a pattern similar to that of the white blood cell count (Fig. S16a). In comparison with other treatment regimens, the observed changes in red blood cell count, monocyte count, and lymphocyte count suggest that the combination of VK-2019 and high-dose GEM may increase hematological toxicity (Figs. 5c, S16). These findings indicate that VK-2019 enhances the hematotoxic effects of GEM in a dose-dependent manner.

The detection results of creatinine and creatine kinase indicated that VK-2019 and GEM did not induce significant renal or cardiac toxicity (Fig. S17), and these findings were further confirmed by histopathological tissue section staining (Fig. S18). Regarding hepatotoxicity, although serum AST levels did not differ significantly across treatment groups, ALT levels indicated that high-dose GEM induced mild hepatotoxic effects, whereas VK-2019 did not exacerbate liver injury (Fig. 5d, e). Histopathological evaluation of liver tissue sections by H&E staining revealed mild cytoplasmic vacuolization in the high-dose GEM group (Fig. S18), further supporting the presence of drug-induced hepatic changes associated with high-dose GEM administration. Histopathological examination of spleen tissue revealed that high-dose GEM induces atrophy of the white pulp, accompanied by a relative expansion of the red pulp, and VK-2019 exacerbates this histological alteration (Fig. 5f). Similarly, bone marrow tissue sections demonstrated comparable findings, with the primary toxicity characterized by vacuolar degeneration of soft tissues within the marrow cavity (Fig. 5g). These pathological changes are considered the principal underlying cause of hematotoxicity associated with the administration of VK-2019 and GEM. Notably, VK-2019 alone elicited discernible toxic effects in both spleen and bone marrow tissues; however, it did not potentiate toxicity when administered concomitantly with low-dose GEM (Fig. 5f, g). In summary, VK-2019 exhibits certain hematotoxic effects; although it may exacerbate the hematotoxicity associated with high-dose GEM, it does not significantly influence the hematotoxic profile of low-dose GEM.

### GEM in combination with VK-2019 shows significant antitumor efficacy in a C666-1 cell-derived xenograft tumor model

**Fig. 6:**
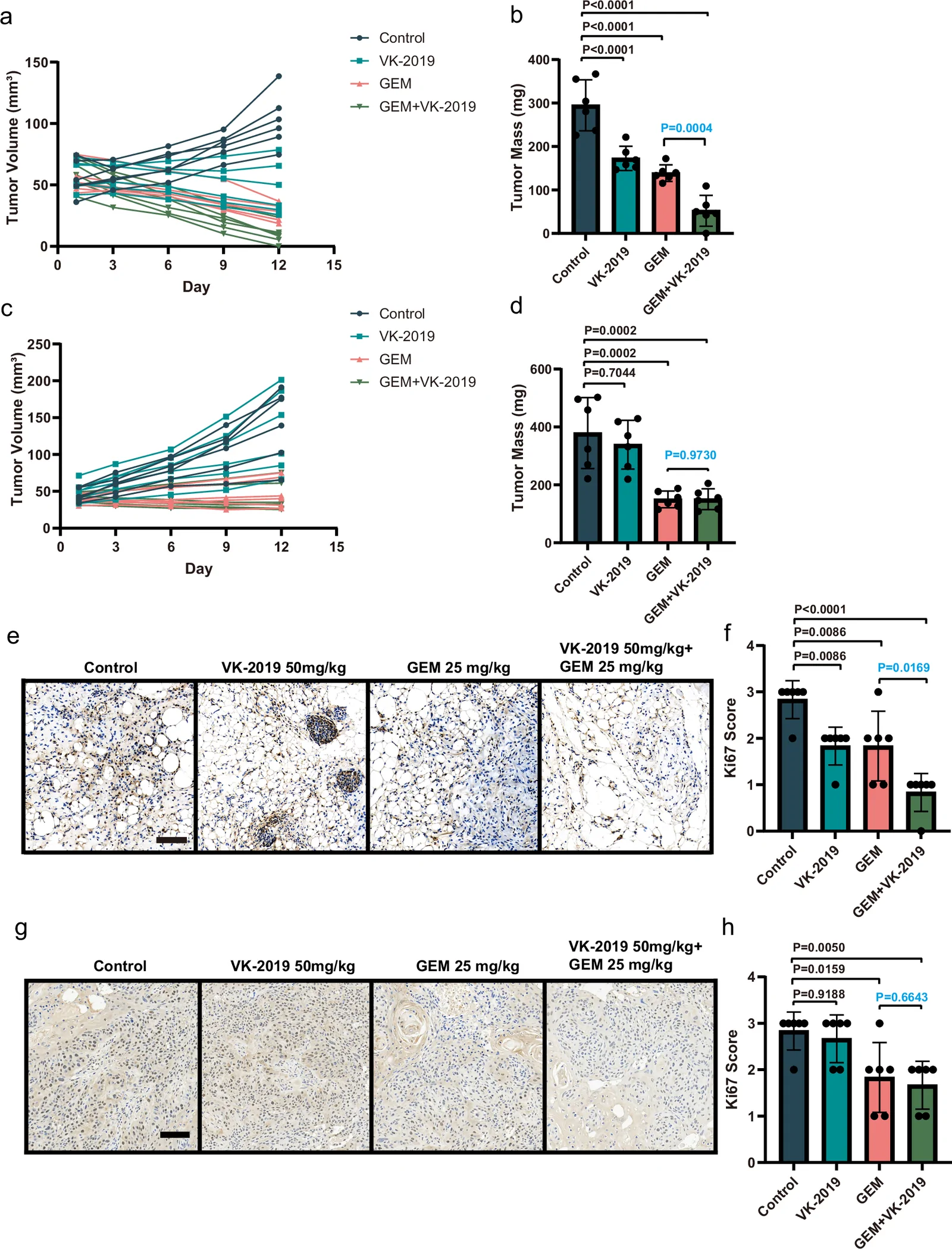
The in vivo anti-tumor effect of VK-2019 combined with gemcitabine. **a** The efficacy curve of VK-2019 combined with GEM in C666-1 xenograft tumor model. One animal in the GEM + VK-2019 group achieved complete tumor regression (CR) by the end of the study. This sample was assigned a value of 0 for weight and volume in the statistical analysis to reflect the maximal therapeutic response. **b** Tumor quality statistics for each medication group on C666-1 xenograft tumors. **c** The efficacy curve of VK-2019 combined with GEM in HK-1 xenograft tumor model. **d** Tumor quality statistics for each medication group on HK-1 xenograft tumors. **e** Ki67 staining on C666-1 xenograft tumors. Scale bars, 100 µm **f** Score statistics of Ki67 staining on C666-1 xenograft tumors. **g** Ki67 staining on HK-1 xenograft tumors. Scale bars, 100 µm. **h** Score statistics of Ki67 staining on HK-1 xenograft tumors. The data are presented as mean ± SD (*n* = 6 biological replicates). Comparisons with the control group were performed using Dunnett’s multiple comparisons test (black *P*-values), and pairwise comparisons between groups were conducted using an unpaired t-test (blue *P*-values).

To evaluate the in vivo therapeutic efficacy of VK-2019 and gemcitabine (GEM), we established xenograft models using EBV-positive (C666-1) and EBV-negative (HK-1) nasopharyngeal carcinoma cells. VK-2019 monotherapy exhibited significant tumor growth inhibition in the C666-1 model (Figs. 6a, b, S19), whereas no appreciable inhibitory effect was observed in the HK-1 model (Figs. 6c, d, S19), confirming its EBV-dependent activity. Notably, the combination of VK-2019 and GEM elicited potent anti-tumor activity in C666-1 xenografts, resulting in marked tumor regression compared to GEM monotherapy; complete tumor regression was observed in one instance (Fig. 6a, b). Conversely, in the HK-1 model, the addition of VK-2019 failed to improve therapeutic outcomes over GEM alone (Fig. 6c, d).

Immunohistochemical analysis of Ki-67 corroborated these findings. The combination therapy significantly reduced the Ki-67 proliferation index in C666-1 tumors compared to the GEM monotherapy group (Fig. 6e, f). Consistent with the tumor volume data, no significant difference in Ki-67 expression was detected between the combination and monotherapy groups in HK-1 xenografts (Fig. 6g, h). Collectively, these results demonstrate that VK-2019 significantly potentiates the in vivo anti-proliferative efficacy of gemcitabine specifically against EBV-positive tumors.

## Discussion

NPC remains a significant clinical challenge, particularly in endemic regions where EBV infection is ubiquitous [3, 31]. In this study, we demonstrate that VK-2019, a specific inhibitor of the viral protein EBNA1, acts as a potent chemosensitizer for GEM in EBV-positive NPC. Our findings reveal that the combination of VK-2019 and GEM significantly potentiates cytotoxicity in vitro and induces tumor regression in vivo, an effect remarkably superior to either monotherapy. Crucially, we identify a novel synergistic mechanism driven by the convergent upregulation of the mitochondrial tumor suppressor PRODH, establishing a distinct therapeutic strategy for EBV-driven malignancies. Mechanistically, our data elucidate a critical axis linking EBNA1 inhibition to metabolic reprogramming. Consistent with reports indicating that VK-2019 down-regulates c-Myc [13], we observed that treatment with VK-2019 leads to a rapid downregulation of c-Myc in EBV-positive cells. Unlike previous studies that focused primarily on disrupting viral replication or episome maintenance, we demonstrate a ~ 30-fold chemosensitization effect driven by a novel metabolic reprogramming mechanism. This provides a robust scientific foundation for the next phase of clinical trials evaluating VK-2019 in combination regimens.

It is important to acknowledge that the current in vivo efficacy studies were conducted in BALB/c-nu mice, which lack functional T cells. While this model allowed us to isolate and confirm the tumor-intrinsic cytotoxicity mediated by the EBNA1-PRODH axis, it does not account for the complex adaptive immune landscape characteristic of EBV-associated NPC. Given that EBNA1 inhibition may modulate tumor-immune crosstalk, further investigations in immunocompetent or humanized mouse models are necessary to fully elucidate the integrated therapeutic potential and immune-mediated toxicities of this combination.

The therapeutic specificity of this approach is underscored by the lack of efficacy in EBV-negative HK-1 models. Since EBNA1 is uniquely expressed in infected cells and lacks a human homolog, VK-2019 functions as a highly targeted agent. However, our in vivo safety assessment indicated that VK-2019 could exacerbate GEM-induced hematologic toxicity. This observation is consistent with clinical findings by Colevas et al. [14], potentially attributable to off-target effects or the intrinsic pharmacological properties of VK-2019. Importantly, our data suggest that dose optimization can effectively mitigate these adverse events while maintaining therapeutic efficacy. These findings suggest a narrow but viable therapeutic window where lower doses of conventional chemotherapy can be potentiated by EBNA1 inhibition, potentially reducing the systemic burden on the hematopoietic system.

In conclusion, VK-2019 sensitizes NPC to gemcitabine by dismantling the EBNA1-c-Myc axis and convergently activating PRODH. These findings provide a molecular rationale for current clinical trials, offering a promising, mechanism-based strategy to overcome chemoresistance in EBV-associated malignancies.

## Materials and methods

### Cell culture

The human NPC cell lines C666-1 (EBV-positive) and HK-1 (EBV-negative) were purchased from Procell. SNU-719, a naturally EBV-positive gastric cancer (EBVaGC) cell line was purchased from Fuheng Biology. Both cell lines have been characterized in previous genomic studies as harboring wild-type *TP53* status [19, 32, 33]. In December 2024, both cell lines underwent short tandem repeat (STR) profiling and PCR-based detection to assess potential mycoplasma contamination. All in vitro experiments were performed using cells within the first 50 passages. C666-1 and HK-1 cells were cultured in RPMI-1640 medium (Gibco, 11875119), supplemented with 1% penicillin-streptomycin (Gibco, 15140148) and 10% South American fetal bovine serum, premium grade (VivaCell, C04001), and maintained at 37 °C in a humidified atmosphere containing 5% CO₂.

### Cell viability assay

Seed 2000 cells per well into a 96-well plate and treat with varying concentrations (0.01, 0.083, 0.83, 4.17, 8.34, 41.7, and 83.4 μM) of test compounds, including VK-2019 (MCE, HY-W725179), cisplatin (CPT, MCE, HY-17394), docetaxel (DOX, MCE, HY-B0011), and gemcitabine (GEM, MCE, HY-17026). Incubate the cells for 48 h at 37 °C with 5% CO₂. For combination treatments, maintain VK-2019 at a fixed concentration of 10 μM. Following incubation, add 10 μL of CCK-8 reagent (Sigma-Aldrich, Cat. No. 96992) to each well and incubate for an additional 2 h at 37 °C. Measure absorbance at 450 nm using a microplate reader (Feyond-A500).

### Caspase 3 activity assay

The activity unit of caspase-3 enzyme was defined according to the manufacturer’s specifications (Chemical): one unit corresponds to the amount of enzyme that catalyzes the hydrolysis of 1.0 nmol of the chromogenic substrate Ac-DEVD-pNA per hour at 37 °C under conditions of substrate limitation [34]. pNA (Beyotime, C1116) was used as the reference standard, and absorbance was measured at 405 nm to generate a standard calibration curve (Fig. S1). A solvent-only sample was used as the negative control, whereas the working concentrations of VK-2019 and gemcitabine were both set at 10 μM. Following a 24-h incubation period, cells were collected and lysed. The detection reagent (Beyotime, C1116) was then added, and samples were incubated at 37 °C for 2 h. Absorbance was subsequently measured at 405 nm using a microplate reader (Feyond-A500).

### Flow cytometry

Seed 2 × 10⁵ cells per well into a six-well plate (Corning, 3516), and administer the treatment after complete cell adhesion. The negative control group was treated with solvent only. The experimental groups were exposed to gemcitabine or VK-2019 at various concentrations for 24 h. Q-VD-OPH was used as a broad-spectrum caspase family inhibitor [21]. Prepare a cell suspension from adherent cells, which were separately collected from dead cells in the culture medium, using trypsin (Gibco, 25200072), and incubate at 37°C for 30 min in culture medium. Centrifuge to remove the supernatant, then resuspend the pellet containing a mixture of dead and adherent cells in pre-cooled phosphate-buffered saline (PBS). Following centrifugation to remove PBS, add 100 µL of 1× Annexin V binding buffer (Beyotime, C1062M). Gently resuspend the cell pellet, then add 5 µL of Annexin V-FITC (Beyotime, C1062M) and 5 µL of propidium iodide (PI) staining solution. Incubate the samples at room temperature in the dark for 15 min. After incubation, resuspend the cells in 400 µL of 1× Annexin V binding buffer and analyze by flow cytometry using an Agilent NovoCyte instrument.

### Transcriptome sequencing

Seed 4 × 10⁵ cells per well into a six-well plate (Corning, 3516), and administer the treatment after complete cell adhesion. The negative control group was treated with solvent only. The experimental groups were exposed to gemcitabine or VK-2019 at 5 μM for 24 h. The total RNA extraction was carried out in accordance with the manufacturer’s instructions provided with the Total RNA Isolation Kit (Vazyme, RC113-01). The extracted RNA samples were submitted to Novogene as RNA solutions for agarose gel electrophoresis, Agilent 2100-based quality assessment, and subsequent sequencing. Upon successful qualification, a sequencing library was constructed, and Illumina sequencing was performed.

Perform quality control and data filtering on fastq files using the Fastqc and Trimmatic software packages, respectively [35, 36]. Sequence alignment was conducted using the STAT software package. The reference genome hg38 (GRCh38.p12) and gene annotation GTF file (GRCh38, version 30, Ensembl release 96) were downloaded from GENCODE (www.gencodegenes.org). Gene expression quantification was performed using the RSEM software package on an Ubuntu 14.04.6 system [37]. Data normalization was performed using the OmicShare platform (https://www.omicshare.com/), which included the calculation of Counts Per Million (CPM) values and the identification of differentially expressed genes based on thresholds of |log2 fold change | ≥ 2 and a Q value < 0.05. Subsequently, KEGG pathway enrichment analysis was conducted, followed by data visualization.

### RT-qPCR

Seed 4 × 10⁵ cells per well into a six-well plate (Corning, 3516), and administer the treatment after complete cell adhesion. The negative control group was treated with solvent only. The experimental groups were exposed to gemcitabine or VK-2019 at 5 μM for 24 h. The total RNA extraction was carried out in accordance with the manufacturer’s instructions provided with the Total RNA Isolation Kit (Vazyme, RC113-01). Total RNA was extracted and reverse transcribed using PrimeScript™ RT Master Mix (Takara, Cat. No. RR036A) according to the manufacturer’s instructions. The reaction mixture was processed in a Mastercycler Nexus X1 PCR system under the following thermal cycling conditions: 37 °C for 15 min and 85 °C for 5 s. Real-time fluorescent quantitative PCR was conducted using the TB Green Premix Ex Taq II kit (Takara, Cat. No. RR820A). Gene-specific primers were synthesized by Sangon Biotech, with detailed sequences provided in Supplementary Table 1S. PCR amplification was performed on an ABI QuantStudio 5 system, following the standard two-step cycling protocol with GAPDH serving as the internal reference gene.

### Western blot

Seed 4 × 10⁵ cells per well into a six-well plate (Corning, 3516), and administer the treatment following complete cell adhesion. The negative control group was treated with solvent alone. The experimental groups were exposed to gemcitabine or VK-2019 at a concentration of 5 μM for 24 h. Cells were harvested using a cell scraper and lysed with RIPA lysis buffer (Beyotime, P0013) supplemented with 1% PMSF (Beyotime, ST506). Following denaturation with an appropriate volume of sample loading buffer (Beyotime, P0015L), proteins were separated by SDS-PAGE (Genscript, M00657) and subsequently transferred onto a PVDF membrane (Bio-Rad, 1620177).

For immunoprecipitation experiments, cells were incubated with VK-2019 at a concentration of 5 μM for 24 h. The negative control group was treated with solvent alone. Cells were harvested using a cell scraper and lysed with RIPA lysis buffer (Beyotime, P0013) supplemented with 1% PMSF (Beyotime, ST506). Subsequently, P53 primary antibody (Proteintech, 10442-1-AP) was added, followed by overnight incubation at 4 °C. Immunoprecipitation was performed in accordance with the manufacturer’s protocol using the Proteintech Immunoprecipitation Kit (PK10008).

The total protein was labeled using No-Stain Protein Labeling Reagent (Thermo Fisher Scientific, A44449), and the PVDF membrane was blocked with blocking buffer (Thermo Fisher Scientific, 37565). Primary antibodies against P53 (Beyotime, AF1162), Phospho-P53 (Ser15) (Proteintech, 80195-1-RR), PRODH (Proteintech, 22980-1-AP), ubiquitin (Proteintech, 10201-2-AP), or c-Myc (Proteintech, 10828-1-AP) were incubated at 37 °C for 1.5 h. HRP-conjugated Goat Anti-Rabbit IgG(H + L) (Proteintech, SA00001-2) and HRP-conjugated Goat Anti-Mouse IgG(H + L) (Proteintech, SA00001-1) were applied as secondary antibodies for chemiluminescent detection. Signal detection was performed using BeyoECL Moonlight (Beyotime, P0018M) on a Bio-Rad ChemiDoc MP imaging system, and protein bands were normalized and quantified using Image Lab 6.0 software [38].

### SiRNA silencing of PRODH gene

The PRODH-specific siRNA was synthesized by Genscript, and the corresponding sequence is provided in Table S2. Cell suspensions were resuspended in Opti-MEM medium (Gibco, 31985070) at a density of 1 × 10⁶ cells/mL, supplemented with 200 nM siRNA, and subjected to electroporation using the Gene Pulser Xcell Electroporation System. Electroporation parameters were set to exponential decay mode with a voltage of 140 V.

### Animal studies

For drug toxicity evaluation, Balb/C mice (Gempharmatech, 5 weeks) were randomly assigned to six experimental groups, with six animals per group (3 female and 3 male mice each). The formulations for gemcitabine and VK-2019 consisted of 5% DMSO (ALADDIN, D103277), 45% PEG300 (ALADDIN, P432446), and 50% PBS, which also served as the vehicle control group. Administration was performed via intraperitoneal injection every other day over a two-week period. Body weights of the mice were recorded throughout the study. At the conclusion of the experiment, mice were anesthetized using isoflurane (RWD, R510-22) and humanely euthanized. Cardiac blood was collected immediately post-euthanasia for subsequent analysis, followed by the excision and harvesting of various tissues and organs.

For in vivo anti-tumor efficacy studies, BALB/c-nu nude mice were subcutaneously inoculated with 5 × 10⁶ tumor cells (C666-1 or HK-1) per mouse. When the average tumor volume reached approximately 50 mm³, the mice were randomly assigned to four groups (*n* = 6 per group, 3 female and 3 male mice each, 5 weeks). This was achieved using a stratified randomization method based on initial tumor volume to ensure that the starting mean volumes were equivalent across all experimental groups. Treatment was administered via intraperitoneal injection every other day for a total of two weeks. The experimental endpoint was defined as either complete tumor regression or tumor volume exceeding 500 mm³.

All animal experiments were conducted in accordance with the guidelines approved by the Institutional Animal Care and Use Committee (IACUC-DWZX-2025-028) of the Experimental Animal Ethics Committee at Fudan University Affiliated Eye, Ear, Nose and Throat Hospital, under whose supervision the study was carried out.

### Statistical analysis

Data are presented as mean ± standard deviation (SD) of at least three independent experiments. In all figures, *n* represents biological replicates. Statistical analyses were performed using GraphPad Prism 10.0. Prior to parametric analysis, data were evaluated for normality using the Shapiro-Wilk test and for equality of variances using the Brown-Forsythe test. For comparisons between two groups, an unpaired two-tailed Student’s t-test was employed. For multiple group comparisons, a one-way analysis of variance (ANOVA) followed by Dunnett’s multiple comparisons test (for comparison against a single control) was used. A *P*-value < 0.05 was considered statistically significant.

## Supplementary information

Original Data

supplement information

supplement table 1

## Data Availability

The data generated in this study are available within the article and its supplementary data files.
