# Supplementary material for: VK-2019 enhances gemcitabine’s antitumor activity in EBV-positive nasopharyngeal carcinoma by upregulating PRODH expression
Source: Cell Death Discov. 2026 Jul 8;12:391. doi: 10.1038/s41420-026-03247-z (PMC13631288; doi:10.1038/s41420-026-03247-z)
Supplement: Supplementary file 1 [file 41420_2026_3247_MOESM1_ESM.docx]

**VK-2019 enhances gemcitabine's antitumor activity in EBV-positive nasopharyngeal carcinoma by upregulating PRODH expression**

Figure 2e (**a**) Western Blot results of PRODH protein in each group. The box shows the location of the destination strip. (**b**) Western Blot results of total protein from **a**.

Figure 4c (**a**) Western Blot results of P53 protein in each group. The box shows the location of the destination strip. (**b**) Western Blot results of total protein from **a**.

Figure 4d (**a**) Western Blot results of Phospho-P53 (Ser15) protein in each group. The box shows the location of the destination strip. (**b**) Western Blot results of total protein from **a**.

Figure 4e (**a**) Western Blot results of P53. (**b**) Western Blot results of ubiquitinated P53.

Figure 4f (**a**) Western Blot results of c-Myc protein in each group. The box shows the location of the destination strip. (**b**) Western Blot results of total protein from **a**.

Figure 4h-4i (**a**) Western Blot results of c-Myc protein in each group. The box shows the location of the destination strip. (**b**) Western Blot results of PRODH protein in each group. The box shows the location of the destination strip.
