# Supplementary material for: VK-2019 enhances gemcitabine’s antitumor activity in EBV-positive nasopharyngeal carcinoma by upregulating PRODH expression
Source: Cell Death Discov. 2026 Jul 8;12:391. doi: 10.1038/s41420-026-03247-z (PMC13631288; doi:10.1038/s41420-026-03247-z)
Supplement: Supplementary file 2 [file 41420_2026_3247_MOESM2_ESM.docx]

**VK-2019 enhances gemcitabine's antitumor activity in EBV-positive nasopharyngeal carcinoma by upregulating PRODH expression**

Content：

[Synergy Analysis	2](#_Toc18013)

[PRODH activity assay	2](#_Toc27822)

[Autophagy assay	2](#_Toc26640)

[P53 transcriptional activity detection	2](#_Toc23453)

[Autophagic Flux Assay	3](#_Toc32206)

[EBNA1 Knockdown and c-Myc and PRODH Overexpression	3](#_Toc22243)

[Immunohistochemistry and Hematoxylin-Eosin staining	3](#_Toc10778)

[Wright's-Giemsa staining	4](#_Toc31938)

[Blood sample detection	4](#_Toc10451)

[Supplementary Table S1	5](#_Toc23992)

[Supplementary Table S2	5](#_Toc25644)

[Figure S1	6](#_Toc12308)

[Figure S2	6](#_Toc640)

[Figure S3	7](#_Toc11522)

[Figure S4	8](#_Toc15258)

[Figure S5	9](#_Toc19240)

[Figure S6	10](#_Toc31083)

[Figure S7	11](#_Toc27408)

[Figure S8	13](#_Toc10052)

[Figure S9	13](#_Toc27630)

[Figure S10	13](#_Toc27662)

[Figure S11	14](#_Toc4995)

[Figure S12	15](#_Toc12055)

[Figure S13	16](#_Toc18988)

[Figure S14	16](#_Toc1132)

[Figure S15	17](#_Toc29792)

[Figure S16	18](#_Toc17623)

[Figure S17	19](#_Toc15659)

[Figure S18	20](#_Toc30456)

[Figure S19	21](#_Toc356)

### Synergy Analysis

To quantitatively evaluate the synergistic effect of VK-2019 and gemcitabine, a 6*6 dose-matrix cell viability assay was conducted. C666-1 cells were treated with various concentration combinations of VK-2019 (0, 3.3, 6.7, 13.3, 26.7, 53.3 μM) and GEM (0, 0.83, 1.7, 3.3, 6.7, 13.3 μM) for 48 hours. Cell inhibition data were analyzed using SynergyFinder 3.0 (https://synergyfinder.org/). Four synergy models, including Zero Interaction Potency (ZIP), Bliss independence, Loewe additivity, and Highest Single Agent (HSA), were employed to calculate synergy scores and generate 3D synergy landscapes.

### PRODH activity assay

Seed 1 × 10^7^ cells per well into a plate (Nest, 704202), and administer the treatment after complete cell adhesion is achieved. The negative control group was treated with solvent only. The experimental groups were exposed to gemcitabine or VK-2019 at a concentration of 5 μM for 24 hours. Samples were prepared and data were collected using the ProDH activity detection kit (Abbkine, KTB1431). Enzyme activity unit definition: One unit of enzyme activity is defined as an absorbance change of 0.005 at 600 nm per minute per mg of tissue protein per mL of reaction system.

### Autophagy assay

Seed 1 × 10⁵ cells per well into a glass-bottom culture plate (Nest, 801006), and administer treatments after complete cell adhesion is achieved. The negative control group was treated with solvent alone. Earle's Balanced Salt Solution (EBSS, Beyotime, C0213) was co-incubated with cells for 1 hour as a positive control. Experimental groups were exposed to gemcitabine or VK-2019 at a concentration of 5 μM for 24 hours. Samples were processed using a Cell Autophagy Staining Detection Kit (Beyotime, C3018S) and analyzed by microplate reader (Feyond-A500) and flow cytometry, respectively.

### P53 transcriptional activity detection

Cell suspensions were resuspended in Opti-MEM medium (Gibco, 31985070) at a density of 1 × 10⁶ cells/mL and supplemented with 20 μg/mL pp53-TA-luc, a luciferase reporter plasmid designed to monitor p53 transcriptional activity (Beyotime, D2223). Electroporation was performed using the Gene Pulser Xcell Electroporation System under square wave conditions (180 V, 2 ms, repeated twice; followed by 30 V, 50 ms, repeated five times). Following electroporation, cells were evenly seeded into a 24-well plate (Nest, 702002) and treated with either gemcitabine or VK-2019 at a final concentration of 5 μM. After incubation for 24 hours, cell viability was assessed using the CCK-8 assay kit (Sigma-Aldrich, Cat. No. 96992). Subsequently, the supernatant was removed, and luminescence intensity was measured using the luciferase assay kit (Beyotime, RG005).

Pharmacological Inhibition of p53: To inhibit p53 transcriptional activity, cells were pre-treated with 20 μM Pifithrin-α (Sigma-Aldrich) for 2 hours prior to the addition of gemcitabine (10 μM). Cells were then incubated for an additional 24 hours before being harvested for protein extraction and Western blot analysis.

### Autophagic Flux Assay

To assess autophagic flux, C666-1 cells were treated with VK-2019 (10 μM) or gemcitabine 10 μM) for 24 hours. The lysosomal inhibitor Bafilomycin A1 (100 nM; Sigma-Aldrich]) was added to the culture medium 4 hours prior to cell harvesting to block the fusion of autophagosomes with lysosomes. Cell lysates were then subjected to Western blot analysis to monitor the turnover of LC3B-II (CST, 2775S) and p62 (Abcam, ab91526) protein levels.

### EBNA1 Knockdown and c-Myc and PRODH Overexpression

Genetic perturbations, including the knockdown of EBNA1 and the overexpression of c-Myc or PRODH, were performed via electroporation using the Bio-Rad Gene Pulser Xcell system (Bio-Rad, Hercules, CA, USA). Briefly, exponentially growing C666-1 cells were harvested and resuspended in electroporation buffer at a final concentration of 1* 10^6^ cells/mL. For each transfection, a 500 μL cell suspension was mixed with either 100 nM of siRNA (NC or siEBNA1, sequences were showed in Supplementary Table S2) or 10 μg of plasmid DNA (Empty Vector, pCDNA3.1-c-Myc-OE, or pCDNA3.1-3*flag-PRODH-OE harboring synonymous mutations, purchased from Miaoling Biology). The mixture was transferred to a 4-mm gap electroporation cuvette and system under square wave conditions (180 V, 2 ms, repeated twice; followed by 30 V, 50 ms, repeated five times). Immediately following the pulse, cells were recovered in pre-warmed complete medium and cultured for 48 hours before undergoing downstream drug treatments or being harvested for protein extraction and functional assays.

### Immunohistochemistry and Hematoxylin-Eosin staining

Tumor specimens were fixed in 4% paraformaldehyde (Biosharp, BL539A) at 4°C for 24 hours prior to paraffin embedding. After embedding, sections were deparaffinized and rehydrated using a graded ethanol series, then rinsed with distilled water. Antigen retrieval was achieved by heating the slides in citrate buffer (Sangon Biotech, E673001) under steam for 20 minutes. To quench endogenous peroxidase activity, tissues were treated with 2.5% hydrogen peroxide. Subsequently, nonspecific binding sites were blocked by incubating the sections with 10% goat serum (Abcam, ab7481) for 30 minutes at room temperature, followed by a wash step with PBS (Gibco, 10010023).

Primary antibody targeting Ki67 (Abcam, ab15580) was diluted 1:100 in a blocking solution composed of 2% casein in bovine serum albumin and applied to the tissue sections, which were then incubated overnight at 4°C. Following this, slides were washed and incubated with secondary antibodies (Invitrogen, 31460) for one hour at ambient temperature. Immunoreactivity was visualized using diaminobenzidine as the chromogenic substrate, and nuclei were counterstained with hematoxylin. Stained sections were subsequently scanned and analyzed using the Olympus Slideview VS200 imaging system. The Ki67 labeling index was scored semiquantitatively: samples with fewer than 10% positive cells received a score of 1; those with 10–50% positive cells were assigned a score of 2; and samples exhibiting more than 50% positively stained cells were given a score of 3.

For hematoxylin and eosin (H&E) staining, organs and tissues were fixed in 4% paraformaldehyde solution (Invitrogen, R37814) at 4°C for 24 hours, then processed and embedded in paraffin blocks (Sigma-Aldrich, P3558). Five-micrometer-thick tissue sections were prepared and stained with H&E using the kit from Beyotime (C0105S), followed by digital scanning with an Olympus Slideview VS200 system.

### Wright's-Giemsa staining

The femurs were entirely removed from BALB/c mice and immersed in 4% paraformaldehyde (Invitrogen, R37814) for 24 hours to ensure proper fixation. Afterward, the specimens underwent decalcification using EDTA-based decalcification solution (Biosharp, BL616B), followed by embedding in paraffin wax to form tissue blocks. Tissue sections with a thickness of 5 μm were obtained, subsequently deparaffinized and rehydrated prior to staining with Wright's-Giemsa dye. Once stained, the slides were mounted with neutral balsam (Sangon Biotech, E675007) and digitally imaged using the Olympus Slideview VS200 slide scanning platform.

### Blood sample detection

The collected blood samples were centrifuged at 3000 revolutions per minute for 20 minutes. Levels of alanine aminotransferase (ALT01; PUREBIO), aspartate aminotransferase (AST01; PUREBIO), creatinine (CR01; PUREBIO), and creatine kinase (SIEMENS, DF38) were measured using specific assay kits on the Atellica CH930 system. Additionally, 50 μL of the blood samples were collected into tubes containing EDTA-2K anticoagulant (Solarbio, G0280), and complete blood cell counts were conducted using an automated hematology analyzer (Mindray, BC-760 CS).

**Supplementary Table 1**. Differentially expressed genes (DEGs) in C666-1 cells treated with VK-2019. (C: Control; V: VK-2019). See the supplementary file.

Supplementary Table S1 Primer sequence.

| Name | 5’->3’ |
| --- | --- |
| PRODH-F | GCAGAGCACAAGGAGATGGA |
| PRODH-R | TGGTATTGCTTGTCCCGCTT |
| P53-F | GAGGTTGGCTCTGACTGTACC |
| P53-R | TCCGTCCCAGTAGATTACCAC |
| GAPDH-F | CTGGGCTACACTGAGCACC |
| GAPDH-R | AAGTGGTCGTTGAGGGCAATG |

Supplementary Table S2 siRNA sequence.

| Name | 5’->3’ |
| --- | --- |
| AS1-PRODH | UGAAUAGCCUCUGUCCUAGdTdT |
| S1-PRODH | CUAGGACAGAGGCUAUUCAdTdT |
| AS2-PRODH | AUUGUCAUAGGCAUCCUUGdTdT |
| S2-PRODH | CAAGGAUGCCUAUGACAAUdTdT |
| AS3-PRODH | AGCCAUUUGGUGAAAGAAGdTdT |
| S3-PRODH | CUUCUUUCACCAAAUGGCUdTdT |
| AS-EBNA1 | GUUCCUUCGUCGGUAGUCCdTdT |
| S-EBNA1 | GGACUACCGACGAAGGAACdTdT |
| AS-NC | AGAAGCUUGACACGUUCGAdTdT |
| S-NC | UUCUCCGAACGUGUCACGUdTdT |

Figure S1 Standard curve of UV absorbance and concentration of *p*NA (n=3).

Figure S2 (**a**) Inhibition curve of VK-2019 combined with Cisplatin (CPT) on HK-1 cells. (**b**) Inhibition curve of VK-2019 combined with Docetaxel (DOX) on HK-1 cells. (**c**) Inhibition curve of VK-2019 combined with Gemcitabine (GEM) on SNU-719 cells. The values shown in the legend are IC50 values (mean ± SD, n=3 biological replicates). The working concentration of VK-2019 is 10 μM. IC50, the half maximal inhibitory concentration(μmol/L), was obtained from GraphPad Prism 10.4.

Figure S3 Quantitative pharmacological synergy analysis of VK-2019 and gemcitabine (GEM) in EBV-positive NPC cells. (**a**) Dose-response matrix illustrating the percentage of inhibition in C666-1 cells treated with a 6 * 6 combination grid of VK-2019 (0, 3.3, 6.7, 13.3, 26.7, and 53.3 μM) and GEM (0, 0.83, 1.7, 3.3, 6.7, and 13.3 μM) . (**b**) 3D synergy landscapes generated using four distinct reference models: Zero Interaction Potency (ZIP), Loewe additivity, Bliss independence, and Highest Single Agent (HSA).

Figure S4 (**a**) Volcano plot of differentially expressed genes between the control and VK-2019 medication groups. (**b**) Volcano plot of differentially expressed genes between the control and GEM medication groups. (**c**) Volcano plot of differentially expressed genes between the control and VK-2019/GEM medication groups.

Figure S5 (**a, b**) Representative Western blot images showing the protein levels of EBNA1 (**a**) and PRODH (**b**) following transfection with negative control (NC) siRNA or EBNA1-targeted siRNA (siEBNA1) in C666-1 cells. Total protein staining was employed as the loading control for normalization. The boxed areas indicate the specific bands quantified for analysis. (**c, d**) Quantitative analysis of the normalized volumes for EBNA1 (**c**) and PRODH (**d**) protein expression. Data are presented as mean ± SD from n=3 biological replicates. Statistical significance was determined using an unpaired two-tailed Student’s t-test.

Figure S6 (**a**) siRNA-mediated knockdown of PRODH mRNA expression in C666-1 detected by QPCR (mean ± SD, n=3 biological replicates). (**b**) Western Blot results of PRODH protein in each group. The box shows the location of the destination strip. (**c**) Western Blot results of total protein from **b**.

Figure S7 (**a**) Representative Western blot confirming the overexpression of PRODH (PRODH-OE) in C666-1 cells compared to the empty vector control. Total protein staining was utilized as the loading control for normalization. (**b, c**) Representative flow cytometry histograms (**b**) and quantitative analysis (**c**) of reactive oxygen species (ROS) levels using DCFH-DA staining. (**d**) Representative flow cytometry plots of Annexin V-FITC/PI staining. Ectopic expression of PRODH resulted in an elevation of the apoptotic cell population compared to the vector group. Data are presented as mean ± SD from n=3 biological replicates. Statistical significance was determined using an unpaired two-tailed Student’s t-test.

Figure S8 (**a, b**) Representative Western blot images of p62 (**a**) and LC3B-I/II (**b**) in C666-1 cells treated with indicated drugs in the presence or absence of Bafilomycin A1 (100 nM). Total protein staining was used as the loading control. (**c, d**) Quantitative analysis of normalized protein volumes for p62 (**c**) and LC3B-II (**d**). Data are presented as mean ± SD from n=3 biological replicates. Statistical significance was assessed by one-way ANOVA followed by Dunnett’s multiple comparisons test (black words) or unpaired two-tailed Student’s t-test (blue words).

Figure S9 (**a**) Western Blot results of PRODH protein in each group. The box shows the location of the destination strip. (**b**) Western Blot results of total protein from **a**.

Figure S10 (**a**) Western Blot results of PRODH. The working concentration of Pifithrin-α is 20 μM, and it is co-incubated with C666-1 cells for two hours before the addition of GEM. (**b**) Quantitative analysis of the normalized volumes for PRODH. Total protein staining was employed as the loading control for normalization. The boxed areas indicate the specific bands quantified for analysis. Data are presented as mean ± SD (n=3 biological replicates). Statistical significance versus the control group was determined by unpaired Student’s t-tests (blue words) or Dunnett’s multiple comparisons tests (black words).

Figure S11 (**a**) Western Blot results of P53 protein in each group. The box shows the location of the destination strip. (**b**) Western Blot results of total protein from **a**.

Figure S12 (**a**) Western Blot results of Phospho-P53 (Ser15) protein in each group. The box shows the location of the destination strip. (**b**) Western Blot results of total protein from **a**.

Figure S13 (**a**) Western Blot results of P53. (**b**) Western Blot results of ubiquitinated P53.

Figure S14 (**a**) Western Blot results of c-Myc protein in each group. The box shows the location of the destination strip. (**b**) Western Blot results of total protein from **a**.

Figure S15 (**a**) Western Blot results of c-Myc protein in each group. The box shows the location of the destination strip. (**b**) Western Blot results of PRODH protein in each group. The box shows the location of the destination strip.

Figure S16 (**a**) Platelet (PLT) count statistics. (**b**) Lymphocyte (LYM) count statistics. (**c**) Mononuclear (MON) cell count statistics. The data are presented as mean ± SD (n=6). Comparisons with the control group were performed using Dunnett's multiple comparisons test (black P-values), and pairwise comparisons between groups were conducted using an unpaired t-test (blue P-values).

Figure S17 (**a**) Creatinine (CRE) concentration detection statistics. (**b**) Creatine kinase (CK) concentration detection statistics. The data are presented as mean ± SD (n=6). Comparisons with the control group were performed using Dunnett's multiple comparisons test (black P-values), and pairwise comparisons between groups were conducted using an unpaired t-test (blue P-values).

Figure S18 H&E staining of bone marrow tissue. Scale bars, 100 µm.

Figure S19 Tumor images. The yellow circle on the left represents the sample of tumor regression..
